# Comparative analysis of the transcriptional responses of five *Leishmania* species to trivalent antimony

**DOI:** 10.1186/s13071-021-04915-y

**Published:** 2021-08-21

**Authors:** Julián Medina, Lissa Cruz-Saavedra, Luz Helena Patiño, Marina Muñoz, Juan David Ramírez

**Affiliations:** Centro de Investigaciones en Microbiología y Biotecnología– UR (CIMBIUR), Facultad de Ciencias Naturales, Universidad del Rosario, Bogotá, Colombia

**Keywords:** *Leishmania*, Transcriptomic profile, Trivalent antimony, Orthologous groups, Pathway reconstruction

## Abstract

**Background:**

Leishmaniasis is a neglected tropical disease caused by several species of *Leishmania*. The resistance phenotype of these parasites depends on the characteristics of each species, which contributes to increased therapeutic failures. Understanding the mechanism used by the parasite to survive under treatment pressure in order to identify potential common and specific therapeutic targets is essential for the control of leishmaniasis. The aim of this study was to investigate the expression profiles and potential shared and specific resistance markers of the main *Leishmania* species of medical importance [subgenus *L.* (*Leishmania*): *L. donovani*, *L. infantum* and *L. amazonensis*; subgenus *L.* (*Viannia*): *L. panamensis* and *L. braziliensis*)] resistant and sensitive to trivalent stibogluconate (Sb^III^).

**Methods:**

We conducted comparative analysis of the transcriptomic profiles (only coding sequences) of lines with experimentally induced resistance to Sb^III^ from biological replicates of five *Leishmania* species available in the databases of four articles based on ortholog attribution. Simultaneously, we carried out functional analysis of ontology and reconstruction of metabolic pathways of the resulting differentially expressed genes (DEGs).

**Results:**

Resistant lines for each species had differential responses in metabolic processes, compound binding, and membrane components concerning their sensitive counterpart. One hundred and thirty-nine metabolic pathways were found, with the three main pathways comprising cysteine and methionine metabolism, glycolysis, and the ribosome. Differentially expressed orthologous genes assigned to species-specific responses predominated, with 899 self-genes. No differentially expressed genes were found in common among the five species. Two common upregulated orthologous genes were found among four species (*L. donovani*, *L. braziliensis, L. amazonensis*, and *L. panamensis*) related to an RNA-binding protein and the NAD(P)H cytochrome-B5-oxidoreductase complex, associated with transcriptional control and de novo synthesis of linoleic acid, critical mechanisms in resistance to antimonials.

**Conclusion:**

Herein, we identified potential species-specific genes related to resistance to Sb^III^. Therefore, we suggest that future studies consider a treatment scheme that is species-specific. Despite the limitations of our study, this is the first approach toward unraveling the pan-genus genetic mechanisms of resistance in leishmaniasis.

**Graphical Abstract:**

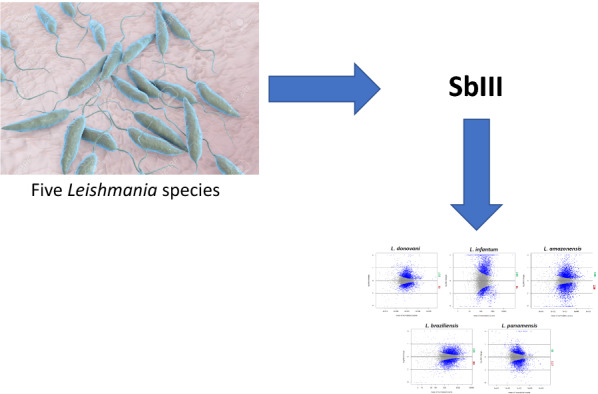

**Supplementary Information:**

The online version contains supplementary material available at 10.1186/s13071-021-04915-y.

## Background

*Leishmania* (Trypanosomatida: Trypanosomatidae) comprises more than 50 species (grouped into five subgenera), at least 20 of which are considered pathogenic for humans [1]. It is the etiological agent of leishmaniasis, a group of tropical infectious diseases transmitted by the bite of the sand fly [2]. It is estimated that 2 million new cases occur annually worldwide, with 70,000 deaths and 350 million people at risk of acquiring the disease [3]. Leishmaniasis, in turn, presents the particularity of a broad spectrum of clinical manifestations that may depend on the *Leishmania* species and/or the host's immune response [4, 5].

*Leishmania* species are classified into subgenera according to propagation within the vector's digestive tract. In *Leishmania* (*Leishmania*), the development occurs in the mid- and foregut, and in *Leishmania* (*Viannia*) in the hindgut [6]. Similarly, both subgenera present a disjunctive geographical distribution pattern: *L*. (*Viannia*) is restricted to the neotropics and *L.* (*Leishmania*) is found in both New World and Old World regions [7]. Its associated clinical manifestations are linked to the specific species of the subgenera. In *L.* (*Leishmania*), we find New World species in the *L. mexicana* complex, where *L. amazonensis* stands out, one of the dominant pathogenic species in Latin America, responsible for diffuse forms of cutaneous leishmaniasis (CL). Here, we also find Old World species, the *L. donovani* complex that causes the visceral form of the disease, with *L. infantum* and *L. donovani*, species with particular endemicity in southern Asia (India, Bangladesh, and Nepal) and north-east Africa (Sudan and Ethiopia) [8, 9]. In *L.* (*Viannia*), *L. braziliensis* and *L. panamensis* stand out, representing the most frequent causes of CL and mucosal leishmaniasis (MCL) in Latin America, especially in Colombia, Ecuador, and Brazil [10].

The number of chromosomes of each species also differs in *L. infantum* and *L. donovani* (36), *L. amazonensis* (34), and *L. braziliensis* and *L. panamensis* (35). This occurs due to the fusion of chromosomes 8 and 39 and 20 and 36 in the *L. mexicana* complex, compared to the *L. major* genome. In the *L. braziliensis* complex, the fusion occurs with chromosomes 20 and 34 [11]. *Leishmania* chromosomes are made up of sizable polycistronic transcription units (PTUs) of functionally unrelated genes. Although not tightly regulated, RNA synthesis has been associated with an accumulation of acetylated histone H3 at the end of the chromosome and in the strand-change regions, marking the start and end of PTU transcription. The presence of a novel modified nucleotide base (β-d-glucopyranosyloxymethyluracil), known as Base J, plays a critical role in ensuring that transcription termination occurs only at the end of each PTU, rather than at the polyadenylation sites of each gene [12–14]. Polycistronic pre-mRNAs are processed into mature trans-spliced mRNAs coupled by a leader RNA and polyadenylation as means of genetic regulation [15].

Around 200 genes are reported with differential distribution between *L. major* and *L. infantum* compared with *L. braziliensis*, where the majority encode for the parasite's survival in the macrophage in addition to a series of unique genes for each of the species [16]. A marker of the subgenus *Leishmania* (*Viannia*) is the RNA interference machinery, which functions as an antiviral defense mechanism against the *Leishmania* RNA virus-1 (LRV1), which seems to impact the virulence of the parasite [17, 18]. Another signature of this subgenus is high expression of molecules such as glycoprotein (GP) GP63 and GP46, known surface components that act on the tropism of CL, development inside the vector, and neutralization of the host macrophage defense system [19–23]. The subgenus *Leishmania* (*Leishmania*) has targets associated with visceral leishmaniasis (VL), such as the genes encoding proteins A2 and 6-phosphogluconate dehydrogenase (6PGDH), besides differential expression of genes such as the catalytic subunit of DNA polymerase A (POLA), heat-shock protein 20 (HSP20), or N-acetylglucosamine-phosphotransferase (NAGT) [24–26].

There is still no vaccine approved for human use; thus chemotherapy and various drugs are the only alternatives for treating the different clinical manifestations of leishmaniasis [27]. These include antibiotics with leishmanicidal action (pentamidine or paromomycin), antitumor treatments (miltefosine), antifungal drugs (imidazoles and triazoles), and essential leishmanicidal drugs (amphotericin B) [28]. Over the past 60 years, antimonials (Sb) (e.g., sodium stibogluconate [SSG] and meglumine antimoniate) have been used as the standard treatment in many countries, mainly due to poor socioeconomic conditions [29–32]. However, with antimonials, an extensive series of therapeutic failures have been reported, associated with multiple factors, including in the patients (adherence to treatment, geographical location, and immune status), the practitioners (failures in the antimonial administration protocols), and the characteristics of the parasite (drug resistance) [32–39].

Multiple studies have focused on determining the mechanisms used by this pathogen to survive under the pressure of antimonials using next-generation sequencing (NGS). Genomic studies have elucidated genetic variations in the number of copies of genes and specific mutations in resistant strains [40–42]. The transcriptomic profile (by RNA-seq) of species exposed to trivalent antimony (Sb^III^) has suggested that *Leishmania* uses differential pathways and global changes in gene expression to reach an adaptive phenotype, as reported in *L. infantum* [43], *L. donovani* [44], *L. amazonensis* [45], *L. panamensis*, and *L. braziliensis* [46]. These and other studies have demonstrated that this behavior generally goes hand in hand with an alteration in the number of copies of particular genes locally or throughout the chromosome [47–49]. Here, the transcripts associated with resistant lines involved a series of metabolic pathways directly associated with drugs and antibiotics or involved in glycolysis and fatty acid processing, in contrast to reducing the expression of factors associated with motility and replicative processes, related to the ribosomal machinery. In addition, some specific genes or processes have been reported as potential resistance targets, including transporters of molecules and metal compounds, heat-shock proteins such as HSP70, and genes encoding for amastins or transmembrane channels such as aquaporin 1 (AQP1) [50, 51]. Other interesting results include a higher number of transcripts associated with resistance to trivalent antimony than other treatments for *L. donovani*. Autophagy induction by *L. amazonensis* represents a survival or cell death strategy or a different expression of genes that encode for some ribosomal proteins whose function in the SSG-resistant *Leishmania* species is still unknown [44, 46, 52, 53].

Factors that may contribute to virulence and pathogenicity have been investigated in different species responsible for the presentations of the disease [54, 55]. Some of these studies also include lines resistant to Sb^III^ and other drugs or focus on factors closely related to treatment, thus establishing the influence of conserved and species-specific traits [56–58]. Despite this, to date, no interspecific comparisons of transcriptomic profiles have been conducted among *Leishmania* species with induced resistance to Sb^III^. Therefore, we studied the expression profiles and potential shared and specific resistance markers of the main *Leishmania* species of medical importance [subgenus *L.* (*Leishmania*): *L. donovani*, *L. infantum*, and *L. amazonensis*; subgenus *L.* (*Viannia*): *L. panamensis* and *L. braziliensis*] resistant to Sb^III^.

## Methods

### Sequence and transcriptome data download

To homogenize the data (RNA-seq reads, stranded, paired) for each of the five *Leishmania* species (*L. donovani*, *L. infantum*, *L. amazonensis*, *L. panamensis*, and *L. braziliensis*), we used the limiting number of replicates of the articles with fewer samples (*L. infantum* and *L. donovani*), and the others were adapted. Then, two biological replicates corresponding to populations with experimentally induced resistance to Sb^III^ and another two to their wild-type counterparts (sensitive to Sb^III^) were used per species (each one composed of two paired RNA-seq reads), available in the European Nucleotide Archive (ENA) databases and National Center for Biotechnology Information (NCBI) in fastq.gz format (https://www.ebi.ac.uk/ena/browser/home) (Additional file 1: Table S1). These were downloaded using GNU Wget software that is part of the application console. Finally, the data were selected, taking as criteria the half maximal effective concentration (EC50), and using promastigotes in the log phase. The quality control of the crude sequences was determined using the FastQC tool, using all parameters except those involving GC content [59]. Subsequently, we removed the adapters previously found with the FastQC Adapter Content report using the CutAdapt tool (https://cutadapt.readthedocs.io/en/stable), and the low-quality reads were removed using Trimmomatic software (TOPHRED33; SLIDINGWINDOW:4:28; MINLEN:36) [60, 61]. Simultaneously, the reference genomes (.fasta) and the annotation files (.gff) of each species were downloaded from the kinetoplastid parasite database, EuPathDB (http://eupathdb.org) (https://tritrypdb.org/tritrypdb/app) [62].

### Differential expression analysis

Initially, the annotated genomes were converted to Gene transfer format (.gtf) using the gffread v0.12.1 package for different gene-specific conventions such as chromosome location [63]. Then STAR v.2.7 software was used to index the reference genomes using the annotation files (.gtf). Finally, the previously mentioned fastq.gz files were aligned, generating a BAM file, again with STAR v.2.7 software (SortedByCoordinate; runThreadN:10; Per-sample 2-pass mapping) [64]. We then used the binary file and the indexed reference genome as input for HTSeq in the generation of a text file with a table containing the count for each gene. Using the intersection parameter of all non-empty sets and “order” options for the paired-end data, four were generated per species, two from wild-type reads and the other two from Sb^III^-resistant reads, counting only the coding sequences (CDS) [65].

The text file served as input for the R package, Bioconductor DESeq2 v.1.30.0, where the differential expression of the genes was evaluated from a binomial distribution model. Here, the wild-type samples served as a point of comparison for samples resistant to Sb^III^, generating a .csv file with the statistical information for the differentially expressed genes (DEGs) [66]. Afterwards, we filtered the sequences, leaving only those genes that encode known proteins, removing hypothetical proteins. This facilitates analyses such as determining orthologous groups and ontology, comparing profiles between species, and constructing tables. To visualize the DEGs, a volcano diagram was constructed showing the average normalized count under the following criteria of statistical significance: a cut-off point of the level of change > 1 or < 1, for the upregulated and downregulated genes, respectively, including a Benjamini–Hochberg test with an adjusted *P*-value < 0.01 [67].

### Determining orthologous groups and ontology

We performed ontology enrichment analysis using the TriTrypDB tools (http://tritrypdb.org) with Fisher’s exact test with *P*-value < 0.05, using the DEG identification numbers (IDs) as an input. The gene ontology terms were classified into three categories (biological processes, cellular components, and molecular functions). These terms were refined in REVIGO (http://revigo.irb.hr/), where redundant terms were cut through the filter of essential genes. Subsequently, a bar diagram of the gene ontology terms with the highest gene count was made using the ggplot2 visualization package [68].

We assigned an orthologous group to each DEG to homogenize the data for later comparison among species using the additional parameters in the search for gene ID in TriTrypDB (http://tritrypdb.org). A bar diagram was constructed using the ggplot2 visualization package for the orthologous genes to illustrate the relationships with the *Leishmania* species. Those that were upregulated and downregulated were included based on the level of change. A heat map of the DEGs for each chromosome distributed among the five species was generated with the R ggplot2 package [68].

### Establishing metabolic pathways

Using a list of DEG IDs (two per state, upregulated and downregulated for each species), FASTA files were generated through the EupathDB TriTryp tool. Each FASTA file (10 total) was uploaded to the Kyoto Encyclopedia of Genes and Genomes (KEGG) Automatic Annotation Server (KAAS) tool, where BLAST was used as the search algorithm and the kinetoplastid data set available in the manually curated KEGG database. Subsequent signaling pathway reconstruction was analyzed under the option "Pathway map" allowing a graphical visualization based on data grouping in the different metabolic pathways. We used Adobe Photoshop CC software to condense the most relevant data obtained in each graph, including the genes of the different species. Then, the pathways where they were involved were chosen (> 11 DEGs) [64]. Additionally, a table was generated that included the metabolic pathways where DEGs were involved and their location and description (Additional file 5: Table S5).

## Results

### DEGs among ***Leishmania*** species resistant to Sb^III^

**Fig. 1:**
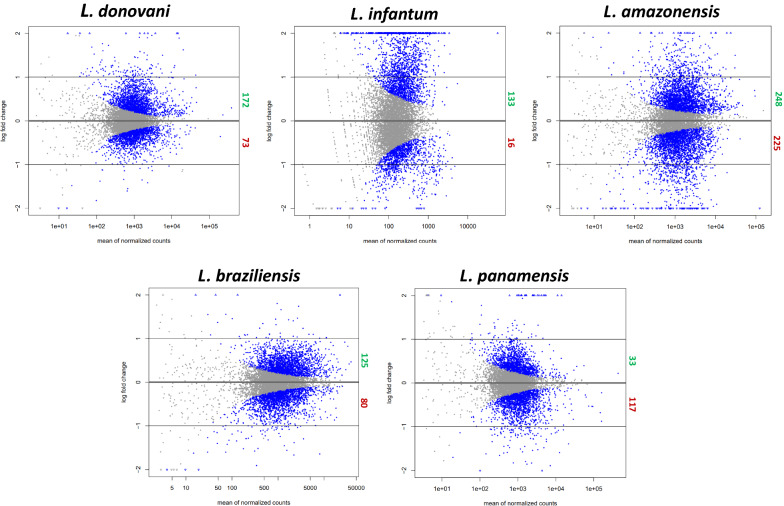
Differential expression between Sb^III^-resistant and Sb^III^-sensitive lines of different *Leishmania* species. Each point represents one gene. Gray dots indicate the genes that were not differentially expressed, and the blue dots, located above and below the black lines (cut-off for the fold change [> 1 and < − 1]), represent differentially expressed genes, adjusted *P*-value < 0.01

**Fig. 2:**
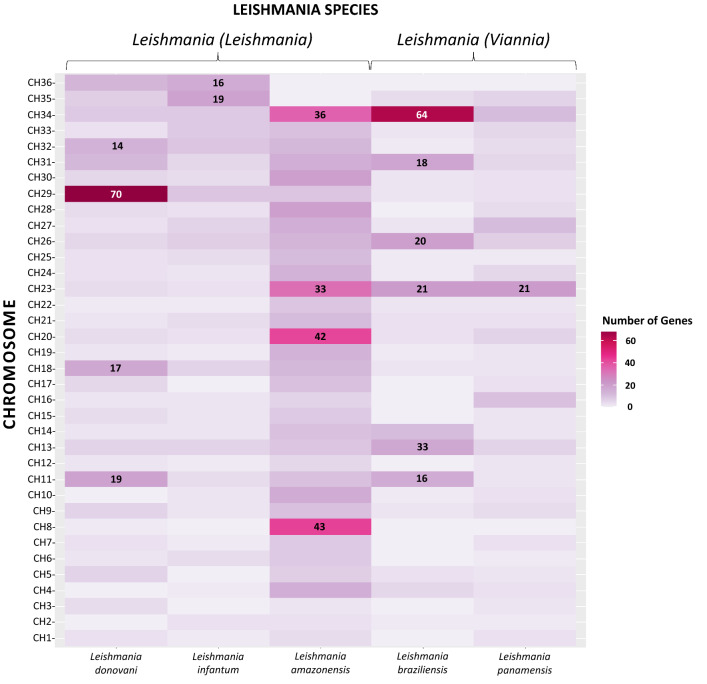
DEG distribution on the chromosomes of *Leishmania* species. Darker colors represent a larger number of genes

No significant differences between the biological replicates of each species were found with their limited sampling. A total of 1222 differentially expressed genes were identified, counting all species (*P*-value < 0.01), of which 711 were upregulated and 511 downregulated. *Leishmania amazonensis* presented the highest number of DEGs in both categories (248 and 225, respectively), while *L. panamensis* and *L. infantum* presented the lowest number of upregulated (33) and downregulated (16) DEGs. In addition, *L. donovani* had 172 and 73 and *L. braziliensis* 125 and 80 DEGs. Genes can be visualized in volcanoes, showing the relationship between mean expression and level of change for each one (Fig. 1; Additional file 2: Table S2; Additional file 3: Table S3). The distribution of the DEGs on the chromosomes of the species belonging to the subgenus *L.* (*Leishmania*) shows a homogeneous pattern for *L. infantum*, a significant number of DEGs on chromosome 29 of *L. donovani* (70), and a considerable number of genes on chromosome 34 for *L. amazonensis* (64). The New World species (*L. amazonensis*, *L. braziliensis*, and *L. panamensis*) also coincide with a high number of DEGs on chromosome 23 (Fig. 2; Additional file 2: Table S2, Additional file 3: Table S3).

### Gene ontology (GO) of DEGs among ***Leishmania*** species resistant to Sb^III^

**Fig. 3:**
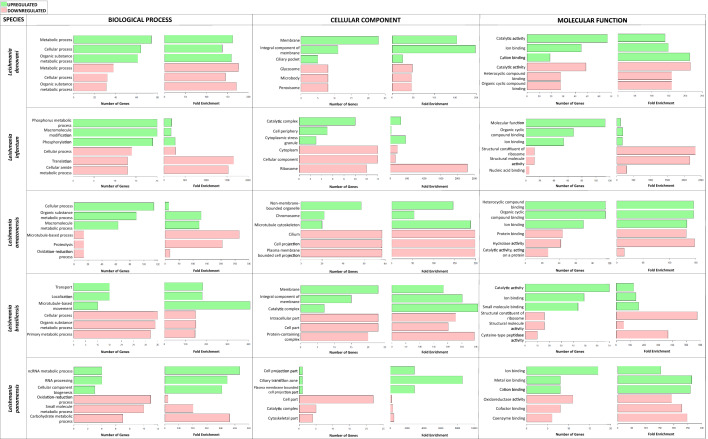
Gene ontology of DEGs among *Leishmania* species resistant to Sb^III^. Green bars represent those in upregulated functional groups and red bars represent those in downregulated groups

Different numbers of DEGs associated with ontological processes were found, as follows, with the first value indicating downregulated processes and the second upregulated: *L. donovani* (137, 128), *L. infantum* (46, 64), *L. amazonensis* (160, 69), *L. braziliensis* (62, 97), and *L. panamensis* (87, 35) (Additional file 4: Table S4). In addition, the prominent roles of the DEGs of the Sb^III^ -resistant populations in each category of GO functional groups were determined (Fig. 3).

A significant number of upregulated genes were observed in the *L.* (*Leishmania*) subgenus related to biological processes. In contrast, the pattern was reversed with the *L.* (*Viannia*) subgenus species, where the downregulated genes were related to different metabolic processes. Antimonial induction affects the metabolism of *Leishmania* (organic substances, drugs, and antibiotics) and cellular processes associated with redox, protein folding, and replication. The group of cellular components was characterized by an increase in the expression of the genes that encode membrane proteins and their integral components and their movement structure. On the other hand, the downregulated DEGs were related to internal components like cytoplasm and ribosome. The most representative upregulated genes with molecular function were associated with catalytic and transport processes and binding to small molecules, metal compounds, and drugs.

The downregulated genes followed a structural pattern, and we found a reduction in protein and nucleic acid binding, hydrolase, and oxidoreductase activity (Additional file 4: Table S4). At the individual level, the gene ontology with the highest enrichment was *L. donovani* (regulation of DNA replication [6899]), *L. infantum* (protein-binding complex [33,193]), *L. amazonensis* (metabolism of isocitrate [2288]), *L. braziliensis* (metabolism of cysteine and glycerol [5267]), and *L. panamensis* (metabolism of sphingolipids [8566]). This also highlights the few upregulated processes associated with the cellular component in these species (Additional file 4: Table S4).

### Comparison of the transcriptomic profiles of *Leishmania* species

**Fig. 4:**
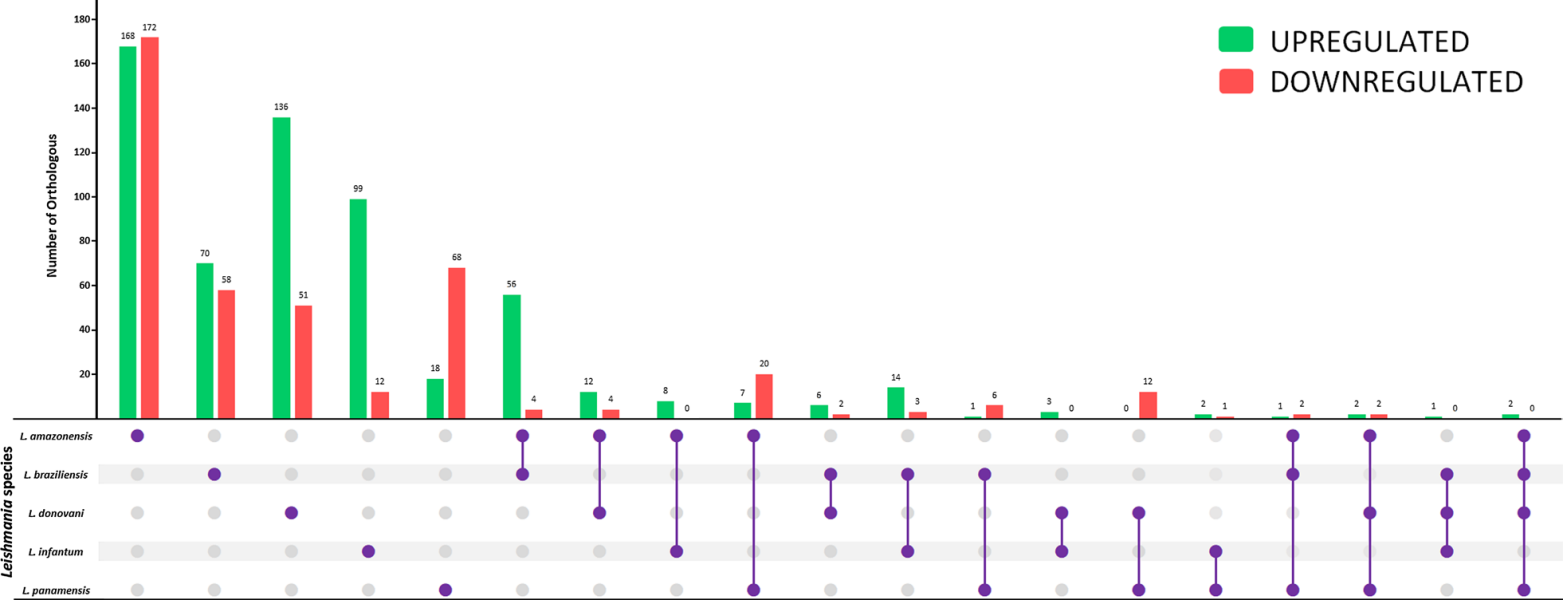
Number of orthologous genes shared and exclusive to each *Leishmania* species (full purple points). Green bars represent those that are upregulated and red bars represent those that are downregulated

The orthologous genes of each species were predominant in proportion to the shared ones. This occurred for both downregulated (red bars) (361) and upregulated (green bars) (491) DEGs. They were followed by the shared genes between any two species (downregulated: 84; upregulated: 141), where each combination between two species had at least one shared ortholog. In *L. donovani* and *L. infantum*, involved in VL, three orthologs were found (OG6_100045, OG6_101028, OG6_101741), related to the transport of substances and catalytic events (Fig. 4, Additional file 2: Table S2).

Shared orthologs were found in three combinations of species. The first, among the subgenus *L.* (*Viannia*) species together with *L. amazonensis*, are distributed in the New World and are generally associated with CL (OG6_156932, OG6_100025, OG6_142640), related to microtubule motor proteins and molecular binding enzymes. Another combination of similarities in orthologs was between *L. amazonensis*, *L. donovani*, and *L. panamensis* (OG6_110995, OG6_127587, OG6_101746, and OG6_157042). Only one common ortholog was found in *L. braziliensis*, *L. donovani*, and *L. infantum* (OG6_119258). Finally, no common orthologs of the subgenus *L.* (*Leishmania*) were found (Fig. 4, Additional file 3: Table S3). No orthologous genes were shared among all species. Only two (OG6_152462 and OG6_200283) were shared among four species (*L. donovani*, *L. braziliensis*, *L. amazonensis*, and *L. panamensis*), both upregulated, associated with an RNA-binding protein and cytochrome b^5^, respectively. A bar plot illustrates the number of shared and self-DEGs of lines resistant to Sb^III^ of *Leishmania* species (Fig. 4). A list of the specific and shared orthologs and the statistical information is also provided (Additional file 2: Table S2; Additional file 3: Table S3).

### Metabolic pathways associated with DEGs of ***Leishmania*** species resistant to Sb^III^

**Fig. 5:**
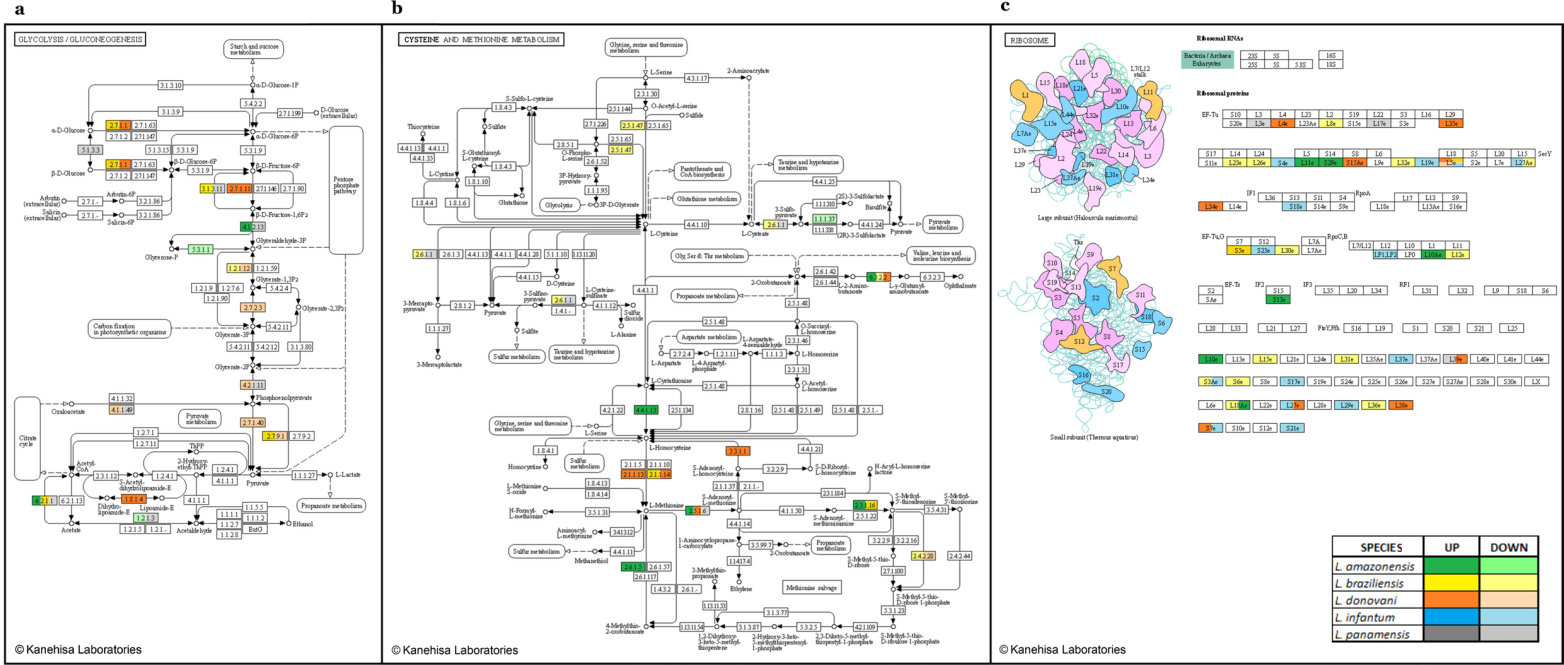
Most representative metabolic pathways for the differentially expressed genes. Glycolysis/gluconeogenesis (ko00010) (**a**), cysteine and methionine metabolism (ko00270) (**b**), and ribosome (ko03010) (**c**)

A total of 139 metabolic pathways were found; 14 had more than 11 DEGs involved, and some were associated with the same component of the pathway. Of these, the three most representative were glycolysis/gluconeogenesis (ko00010) (26), cysteine and methionine (ko00270) (21), and ribosome (ko03010) (54) (Fig. 5a–c respectively). In these cases, differential responses were found between species, even in the same gene (e.g., acetyl-CoA synthetase in the glycolysis/gluconeogenesis pathway was upregulated in *L. braziliensis* but downregulated in *L. panamensis*). On the other hand, referring directly to resistance to treatments and pathology, we found signaling pathways of the metabolism of drugs and other enzymes (ko00983), resistance to antifolates (ko01523), resistance to platinum drugs (ko01524), and leishmaniasis (ko05140) (Additional file 5: Table S5).

## Discussion

Here, we identified a large number (1222) of DEGs between sensitive and resistant lines in the studied *Leishmania* species (Fig. 1). This is possibly a consequence of the basic properties of the parasite, starting with the repertoire of unique genes and the expression patterns that impact its virulence, which do not necessarily have to be exclusively linked to resistance to treatments. Furthermore, *Leishmania* is exposed to other stressful conditions (e.g., oxidative stress in macrophages or contamination by arsenic in the environment), so the resistance mechanisms could be repeated in both situations [69–71]. Both repertoires of DEGs could be discriminated in future studies using cell lines involved during the infection process of the parasite without the treatment intervention. It is worth mentioning that herein we employed sequence data from different studies, and these could be subject to heterogeneous experimental conditions with regard to their conservation and manipulation in methodological processes not defined in the articles, as well as subsequent filtering of the raw sequences, a concerning limitation that we want to highlight. Additionally, we suggest the use of amastigotes in the future when treating the parasite's infective stage and underexposure from the treatment in a real case [72].

In species lacking transcriptional regulation such as *Leishmania*, one of the adaptive mechanisms to modulate the gene dose is the change in the number of chromosome copies, whose base number is a chromosomal mosaic where monosomies (like chromosome 2 in *L. major*) to supernumerary chromosomes (like chromosome 5 in *L. major*) can occur [73, 74]. Nevertheless, we cannot be sure that the change in chromosomal somy is directly responsible for the resistance phenotype [75]. In addition, there may be changes associated with more specific processes, such as the variation in the number of copies of regions or even variants generated at the nucleotide level [46, 76]. *Leishmania* can amplify and remove some loci regions through rearrangement and recombination events as a strategy against environmental changes [77, 78]. These could be some of the reasons behind the overrepresentation of DEGs at chromosome 23 in New World species and the relationship to CL (*L. amazonensis*, *L. braziliensis*, and *L. panamensis*) (Fig. 2).

A large proportion of the genes correspond to ABC transporters involved in the entry and distribution of the drug in the parasite cell [79, 80], which agrees with previous findings in *L. infantum* and *L. major*. This points to a potential biomarker of resistance beyond the pathogen's clinical manifestation and geographical distribution [81, 82], but future mechanistic studies are needed to confirm this hypothesis. Chromosome 34 of *L. amazonensis* and *L. braziliensis* has genes related to arginine metabolism. This pathway contributes to the maintenance of the reduction–oxidation balance and the expression of virulence factors such as GP63 and other genes that encode for amastins, the latter of which is vital in the differentiation of *Leishmania* [83], suggesting the need to understand the effect of these genes in the resistance phenotype.

We found GO terms associated mainly with metabolic processes, probably due to the inhibitory action on glycolysis or oxidation of fatty acids by trivalent antimony. This is consistent with previous studies in *L. donovani* and *L. major* (Fig. 3; Additional file 4: Table S4) [84–86]. Regarding molecular functions, we observed the gene ontology associated with binding to different compounds, especially those with ionic properties, probably related to metal-thiol conjugate sequestration by ATP binding cassettes (ABC transporters), a resistance mechanism widely reported in *L. infantum* and *L. major* [87–89]. For the structural components of the pathogen, the most significant was the membrane, likely due to properties such as the number of ergosterols required for protection against reactive oxygen species (ROS) generated in the biological activation of antimony and/or the presence of transporters [90, 91]. In future studies, each of these specific processes could be explored, seeking to inhibit or reduce the impact of treatment actions that could directly and/or indirectly interfere, setting up their role in the parasite's virulence and survival rate, including studies in vivo if possible.

As some product descriptions were redundant even after REVIGO scrubbing, we performed a signaling pathway reconstruction as a supplemental functional analysis. Three pathways stood out for the number of DEGs involved. The first was glycolysis/gluconeogenesis, the main basis of *Leishmania* promastigote metabolism. The latter is also considered a virulence factor, as it facilitates replication in the phagolysosome of macrophages and the generation of typical lesions in mice [92, 93] (Fig. 5a; Additional file 5: Table S5). Glucose transporters and fructose-1,6-bisphosphate, as gluconeogenic enzymes, have been validated as pharmacological flanks; in turn, this pathway indirectly helps to synthesize other flanks, such as mannan from hexoses [94]. The cysteine and methionine pathway has been found to be modulated in *L. donovani* when exposed to amphotericin B and oxidative stress in general. It also contributes to apoptosis inhibition, one of the mechanisms of action of miltefosine [95, 96] (Fig. 5b; Additional file 5: Table S5). Finally, studies related to the ribosome have found differential expression of the proteins of the large subunit (e.g., L23a) involved in translation and with a role in kinase activity in *Leishmania* resistant to pentavalent antimonials, miltefosine, and amphotericin [97, 98]. Likewise, ribosomal proteins play a role in cell growth and apoptosis control [99] (Fig. 5c; Additional file 5: Table S5). Also, in trypanosomatids, regulation of proteins in a specific subcellular compartment, such as ribosomes, is usually exponential in stages of parasite development such as early metacyclogenesis (eg., L5, L11, Rpf2, and Rrs1), showing its importance in translation repression, interaction with specific essential proteins, and its susceptibility to conditions such as oxidative stress [100–103].

Signaling pathway reconstruction showed a more detailed picture of the processes associated with GO terms, where agreement with reports of resistance and participation from biological processes, molecular functions, and cellular components are maintained. Some molecular targets involved in previously studied resistance mechanisms such as multidrug resistance-related protein A (MRPA) or key enzymes in trypanothione synthesis (e.g., gamma-glutamylcysteine synthase and spermidine) were differentially regulated (Additional file 3: Table S3; Additional file 5: Table S5). MRPA contributes to drug transport in the sequestration of Sb^III^–trypanothione complexes in compartments close to the flagellar pocket of the parasite [88]. On the other hand, trypanothione acts as a nonenzymatic antiparasitic reducing agent, counteracting the oxidative effects of antimonial components, and even preventing their action when conjugated with them [104]. However, we did not find differential expression of aquaglyceroporin (AQP1), a fundamental element in resistance to antimony mediated by drug entry [105]. Further proteomic studies might be conducted to investigate the impact between RNA-seq data and protein expression.

When comparing orthologous groups, we found two shared orthologs between species of both subgenera (*L. amazonensis*, *L. braziliensis*, *L. donovani*, and *L. panamensis*) (Fig. 5, Additional file 3: Table S3). OG6_152462, encoding for RNA-binding proteins (RBPs), was present in the post-transcriptional control processes of gene expression, something fundamental in trypanosomatids. It has been described as a therapeutic target of interest, as it affects the parasite's virulence because of altered expression [106, 107]. The other ortholog was OG6_200283, encoding for a component of the complex NAD(P)H-cytochrome-B5 oxidoreductase, whose upregulation can favor the survival of the parasite in a medium with lipid deficiency, something that it experiences not only when exposed to trivalent antimony but also during its life cycle. It is common in several species, as it participates in the de novo synthesis of linolenic acid, the most common unsaturated fatty acid in *Leishmania*, related to a decrease in oxidative stress ROS [108].

Our results show a more significant proportion of orthologs unique to each species than those shared between two or more species. At the subgenus level, there is no evidence of several significant orthologous relationships: within *L*. (*Leishmania*), there are no proper orthologs, and in *L.* (*Viannia*), only seven good orthologous genes are found (Fig. 4). This is probably because the mechanism of antimony resistance is complex and multifactorial, where the characteristics of each species can contribute to the final response, which reflects the taxonomic classification, the geographical location, and the clinical manifestation. However, a population-level analysis would provide further information [109–111]. On the other hand, some of these orthologs may be associated with a general stress response, and others with the resistance mechanisms of *Leishmania* to antimony, where resistant strains could modify the expected behavior of the metabolic pathways to elaborate their phenotype [112]. This goes together with the different mechanisms of action that the drug must have, including the fragmentation of genetic material, oxidative stress induction, interference in protein interactions, or an increase in intracellular Ca+ [113–115]. Mechanistic studies are needed to respond to these questions and fully understand the concept of resistance that seems species-specific.

Possible limitations of our methodology include the exclusion of non-coding regions (UTR) in the count of gene reads, which provide heterogeneity in transcription by regulating the RNA stability and/or the efficiency of translation [116, 117]. Limitations in functional annotation are inherent in one or several species. Also, there is remarkable specificity of orthologous groups, where two or more genes belong to the same metabolic pathway and/or molecular/structural process [118]. For example, the orthologs OG6_101492 and OG6_101499 both putatively encode for the structural maintenance of the chromosome in *L. amazonensis*, but one of them belongs to a protein family (Additional file 2: Table S2). Using only the sequence of two biological replicates for each condition can also have repercussions in generating potential deficiencies in sensitivity and statistical specificity or the absence of data on other differentially expressed genes; thus, additional validations are required. Another scenario that may contribute to a higher proportion of self-genes per species is that the ortholog is shared, but its differential expression is different, as occurs, for example, with OG6_119258, where this nucleobase transporter is downregulated in *L. braziliensis*, *L. donovani*, and *L. infantum*, but upregulated in *L. panamensis* (Additional file 2: Table S2; Additional file 3: Table S3). These changes in the transcriptome could also be correlated to alternative trans-splicing, a mechanism of heterogeneity described in trypanosomatids such as *L. major* and *T. brucei* [119, 120]. Also, changes may occur at the transcript level due to differences in mRNA maturation and stability mediated mainly by polyadenylation or deadenylation events, mainly by RNA-binding proteins [121].

The use of promastigotes instead of amastigotes must also be considered a limitation of the current study. Transcriptomic differences have been demonstrated in both stages, and amastigotes, known as the infective and intracellular stage of the parasite, represent the best alternative in in vitro drug exposure studies [72]. Experimental conditions that are likely to affect the results include the stability of the sensitive and resistant lines determined by their respective media. It is worth mentioning that the resistance selection rounds and their duration also varied depending on the species, but this went hand in hand with the necessary doses until reaching a common cut-off point (EC50). It should also be noted that resistance and other virulence factors can vary even intraspecifically. Thus, future studies should consider more than just coding sequences and should consider a more significant number of strains of each of the species evaluated in this work, preferably collected from common geographical areas [122, 123]. Establishing relationships between our results and those of studies carried out at the proteome and chromosomal structure level (general and local) could contribute to a better understanding of resistance mechanisms across species.

In addition, future studies should consider the role of hypothetical proteins and genes with unknown functions, as they represent a significant percentage of potential individual markers. Despite the limitations highlighted herein, this study is the first to provide a comprehensive comparative analysis between several species of different subgenera, providing a functional perspective of general (ontology) and specific (reconstruction of signaling pathways) forms, contributing to the basic knowledge necessary for the development of potential treatments based on epidemiology and the behavior of this parasite resistance linked to a public health problem.

## Conclusions

Our study contributes to a better understanding of the trivalent antimony resistance patterns of various *Leishmania* species. We found that there was a species-specific response predominance, mainly associated with biological processes and cellular components. Likewise, some probable potential DEGs were evidenced that could be evaluated as therapeutic targets directed to a specific subgenus or clinical manifestation, which based on their function could be regulated in stressful conditions beyond exposure to Sb^III^, such as lipid deficiency and post-transcriptional control (RNA and cytochrome B5 binding protein). Furthermore, we highlight some limitations of the NGS approach in determining patterns of resistance mechanisms among different *Leishmania* species, and therefore the proposal of treatment strategies (species-specific or a holistic response), as well as other variables that should be considered for future research in this field.

## Supplementary Information

**Additional file 1: Table S1.** General information about the samples and reference genomes used in this study. Samples were obtained from the European Nucleotide Archive (ENA) and reference genomes from TriTrypDB.

**Additional file 2: Table S2.** Shared orthologous shared between *Leishmania* species.

**Additional file 3: Table S3.** Exclusive orthologs of *Leishmania* species.

**Additional file 4: Table S4.** Gene ontology (three functional groups: cellular component, molecular function, and biological process) of *Leishmania* species.

**Additional file 5: Table S5.** Metabolic pathways and differentially expressed genes involved among *Leishmania* species.

## Data Availability

The data sets used and/or analyzed during the current study are available in Additional file 1: Table S1.

## Abbreviations

DEG: Differentially expressed genes
Sb^III^: Trivalent antimony
CL, VL, MCL: Cutaneous, visceral, mucosal leishmaniasis
PTUs: Polycistronic transcription units
LRV1: *Leishmania* RNA virus-1
GP63, GP46: Glycoprotein 63, 46
HSP70, HSP20: Heat-shock protein 70, 20
6PGDH: 6-Phosphogluconate dehydrogenase
POLA: DNA polymerase A
NAGT: N-acetylglucosamine phosphotransferase
AQP1: Aquaporin 1
EC_50_: Half maximal effective mean concentration
ROS: Reactive oxygen species
CDS: Coding sequence
